# Supplementary material for: Single-Cell Analysis of Neuroinflammatory Responses Following Intracranial Injection of G-Deleted Rabies Viruses
Source: Front Cell Neurosci. 2020 Mar 20;14:65. doi: 10.3389/fncel.2020.00065 (PMC7098990; doi:10.3389/fncel.2020.00065)
Supplement: Supplementary file 1 [file Data_Sheet_1.pdf]

Supplementary Material

Single-cell analysis of antiviral neuroinflammatory responses following intracranial injection of G-deleted rabies viruses

Kee Wui Huang[1] and Bernardo L. Sabatini[1]*

[1] Howard Hughes Medical Institute, Department of Neurobiology, Harvard Medical School, Boston, MA 02115, USA.

* Correspondence:
Bernardo L. Sabatini
bsabatini@hms.harvard.edu

Supplementary Figure S1

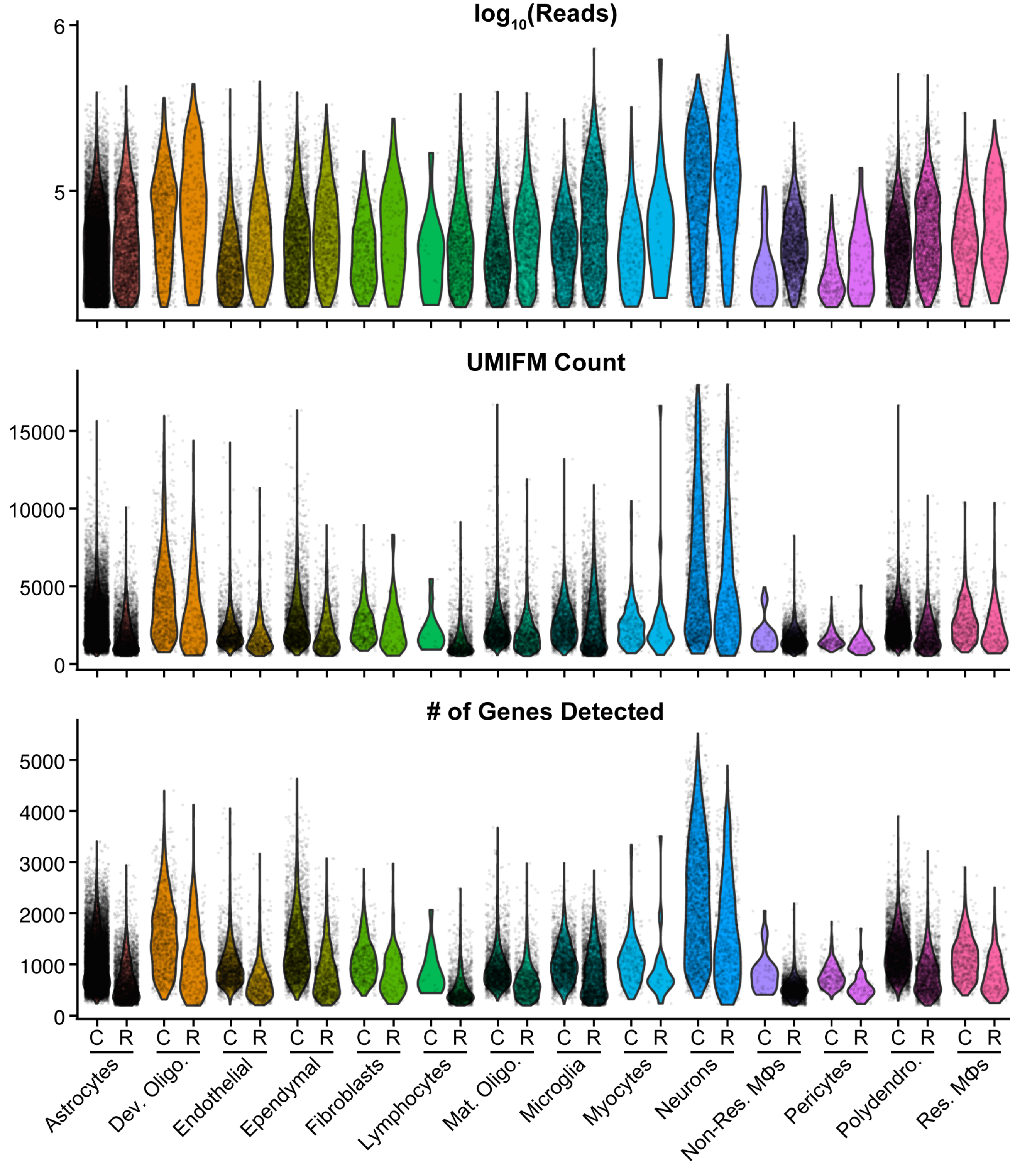

Supplementary Figure S1. Read/UMI count and gene detection rates by cell types. Violin plots showing the log_{10}-transformed read counts (top), UMIFM counts (middle), and number of genes detected (bottom) per cell. Points represent individual cells, and cell type groups are split into either Control (C columns) or RbV-injected (R columns) conditions. Colored areas represent the probability distribution for each group normalized by the number of cells in each group.

Supplementary Figure S2

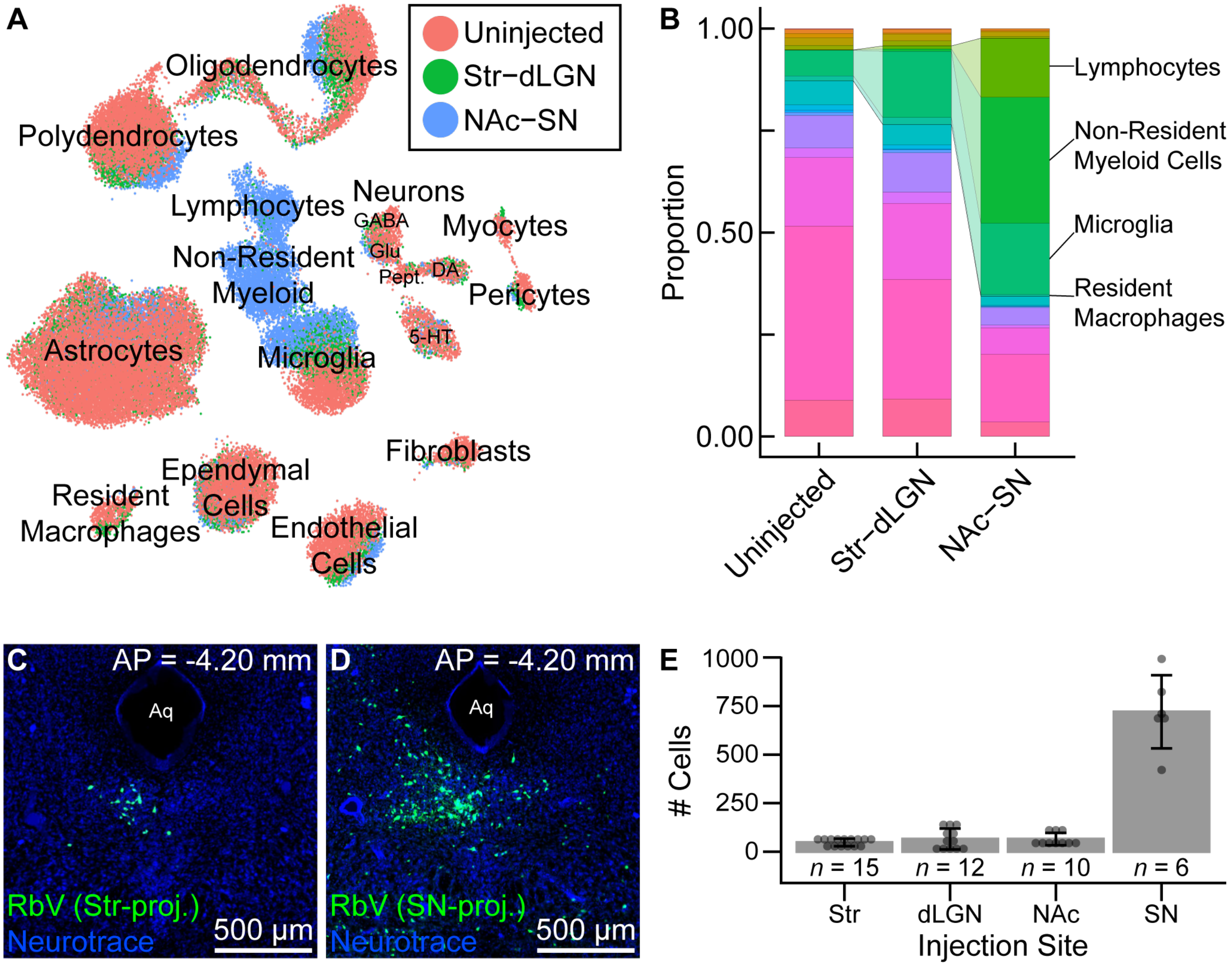

Supplementary Figure S2. Leukocyte recruitment varies with infection magnitude. (A) UMAP plot of merged dataset with each cell color-coded by the injection site grouping. Specific neuron types are indicated and labeled by their primary neurotransmitter. **(B)** Stacked bar plot showing the relative proportion of each cell class/type in each of the injection site groups. **(C–D)** Fluorescence image of a representative coronal section showing neurons in the DRN and surrounding ventrolateral periaqueductal gray infected by RbV injected into the ipsilateral Str (**C**) or SN (**D**). All injections sites were targeted in the right hemisphere, and AP distances are relative to Bregma with negative values indicating positions posterior to Bregma. Injection volumes and virus titers were matched across injection sites. Abbreviations: Aq – cerebral aqueduct. dLGN – dorsal lateral geniculate nucleus. NAc – nucleus accumbens. SN – substantia nigra. Str – striatum. Scale bars: 500 µm. All coronal sections were counterstained with Neurotrace 435. **(E)** Bar plots showing the mean number RbV labeled cells for different injection sites. Counts from individual mice are represented as points, and the height of each bar indicates the mean for each group. Error bars are S.E.M. and *n* indicates the number of mice per group.

Supplementary Figure S3

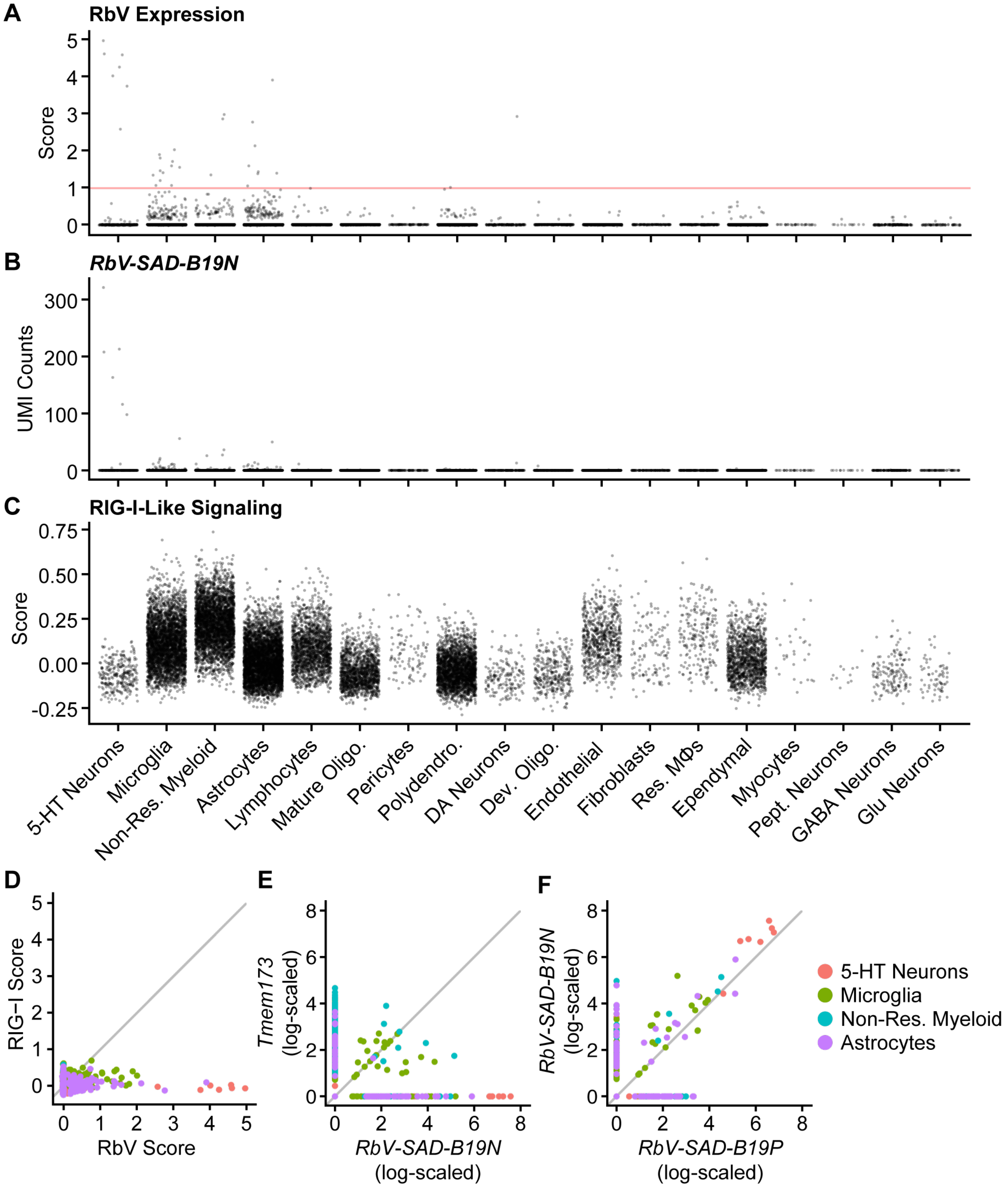

Supplementary Figure S3. RbV transcripts are detected in neurons and phagocytic cells. (A–C) Dot plots showing the RbV gene set expression scores (**A**), *RbV-SAD-B19N* transcript counts (**B**), and RIG-I-Like signaling gene set expression scores (**C**) for individual cells grouped by cell class/type. Only cells from the RbV group are displayed. Cell classes/types (columns) are ordered horizontally by the average RbV expression score. 5-HT neurons have the highest RbV expression scores, followed by microglia, non-resident myeloid cells, and astrocytes. Score threshold is denoted by the horizontal red line. **(D–F)** Scatter plots for gene set expression scores (**D**) and log-scaled gene expression (**E**, **F**) in cells from the four cell types with the highest average RbV gene set expression scores. Points are single cells color-coded by cell class/type. No correlation was observed between the RIG-I-like signaling gene set expression score and RbV gene set expression score (**D**). Expression of *Tmem173* (STING) was highest in non-resident myeloid and microglia, but was low in 5-HT neurons even in cells with high expression of the *RbV-SAD-B19N* (**E**). Expression of genes within the RbV gene set, such as *RbV-SAD-B19N* and *RbV-SAD-B19P* were correlated (**F**). *RbV-SAD-B19N* expression was also higher compared to all other RbV genes, as previously described. Gray lines in D-F indicate the line of unity ($x = y$).

Supplementary Figure S4

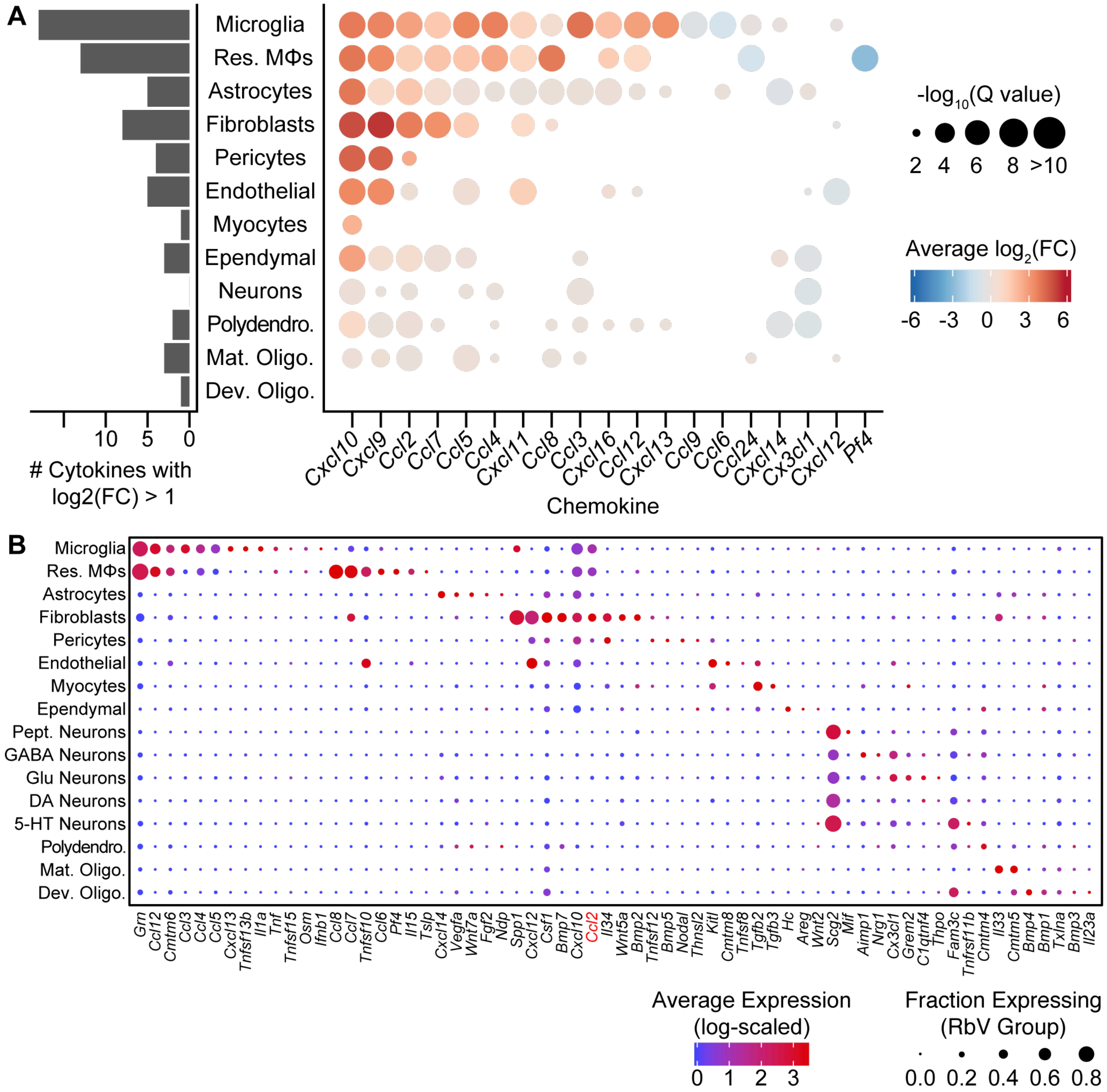

Supplementary Figure S4. Cell type-specific expression of cytokines. (A) *Left*, Bar plot showing the number of significantly up-regulated cytokines (average $\log_2$ fold-change > 1) in each resident cell type. *Right,* Dot plot showing the cell type-specific changes in differentially expressed chemokines. The color of each dot represents the average $\log_2$ fold-change for each cell type cluster, and the size of the dot is scaled by the negative $\log_{10}$ of the adjusted P values (Q values, Benjamini-Hochberg correction). Points in which a gene was not considered differentially expressed for a given cell type (Q value ≥ 0.01) are not displayed. **(B)** Dot plot showing expression of selected cytokine genes (columns) by cell type (rows). The color of each dot represents the average log-scaled expression of each gene in a given cell type, and the size of each dot represents the fraction of cells of that type in which transcripts for that gene were detected.

Supplementary Figure S5

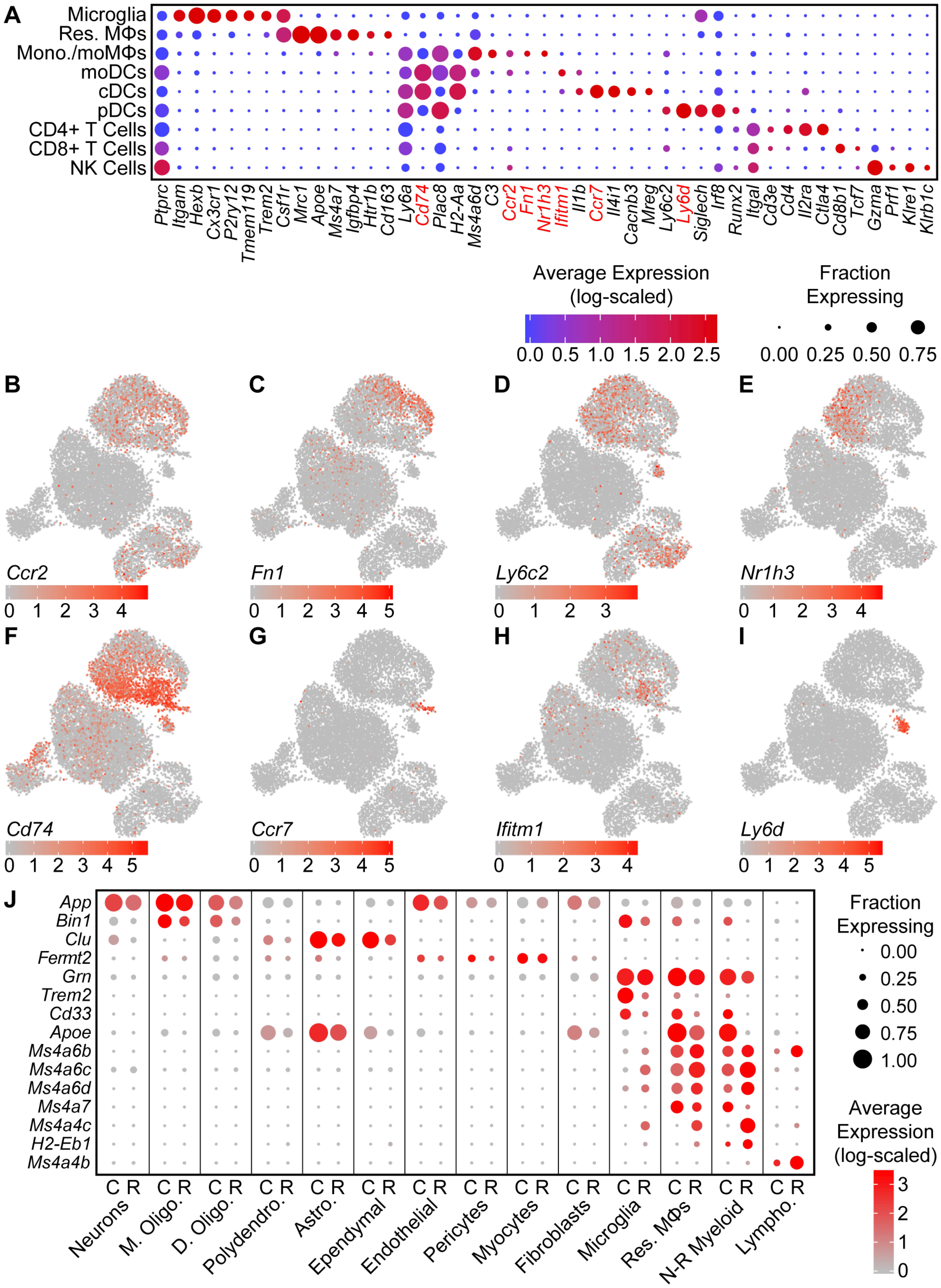

Supplementary Figure S5. Identification of immune cell clusters. (A) Dot plot showing expression of several example genes (rows) used for identifying immune cell clusters (columns). **(B–I)** UMAP feature plots showing the expression of genes that are differentially expressed between non-resident myeloid cell types. Each cell is color-coded by its log-scaled expression for the indicated gene. **(J)** Paired dot plot showing expression of several genes associated with Alzheimer's Disease (rows), separating by cell type (columns) and condition (sub-columns: C – Control; R – RbV).

Supplementary Figure S6

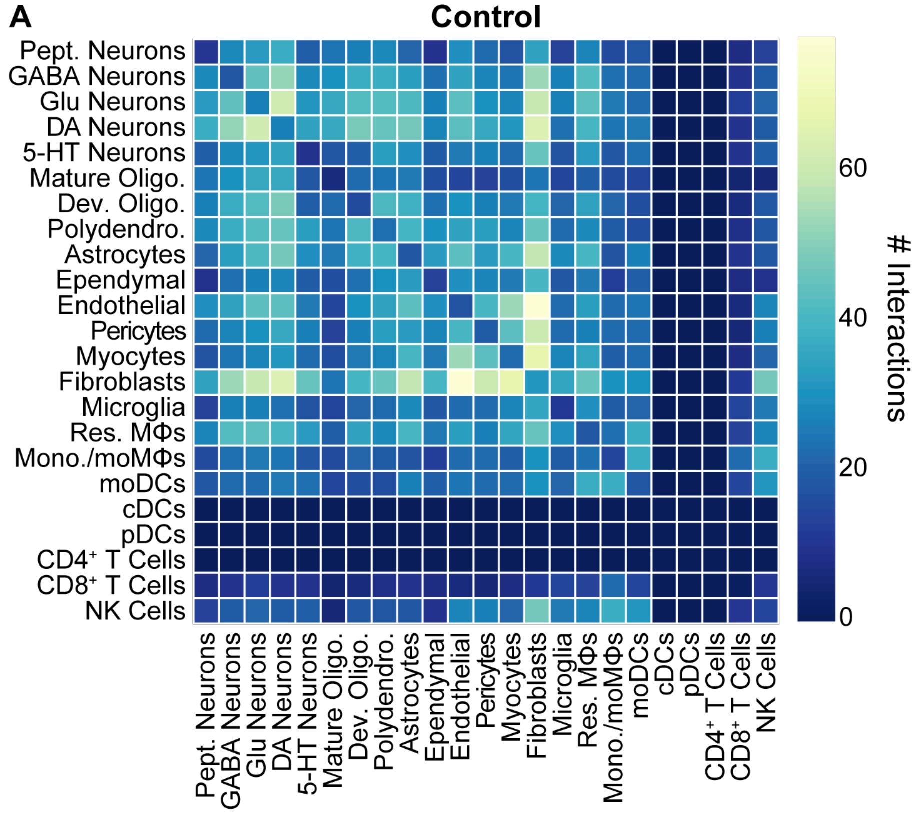

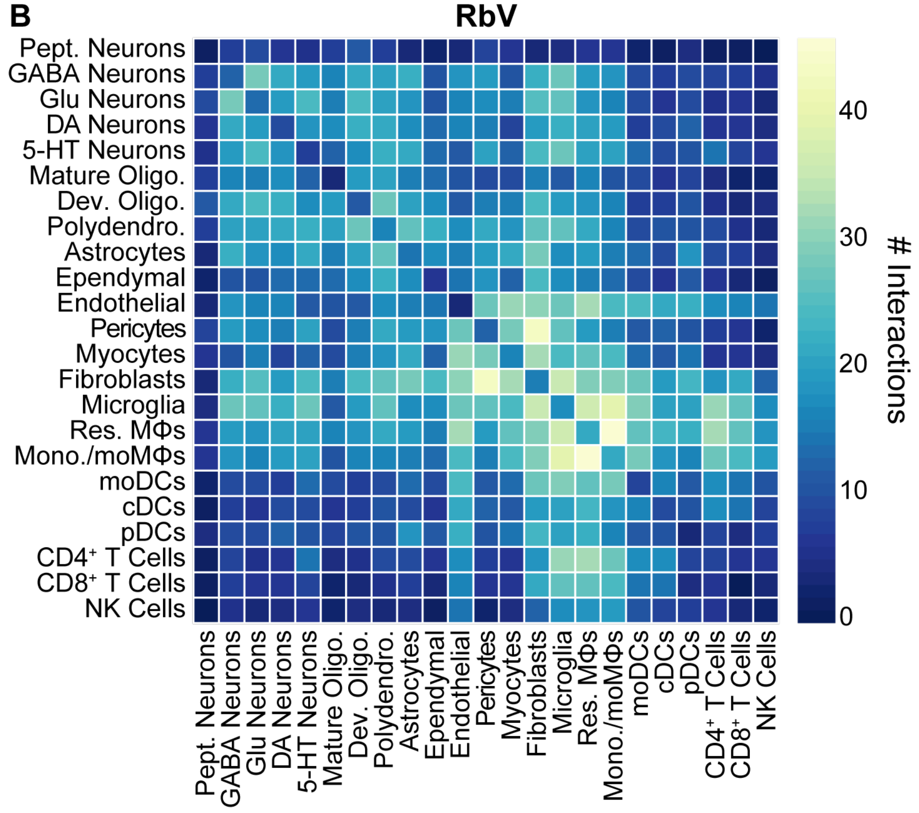

Supplementary Figure S6. Number of inferred interactions between cell types by condition. (A) Heatmap showing the number of inferred interactions between cell types in the control group. The color range is scaled to the maximum number of interactions between a pair of cell types in the Control group. Interaction counts are non-directional, and not weighted by cell type abundance. Interactions for cell types not present in the Control group (e.g. dendritic cells) are at 0. **(B)** Heatmap showing the number of inferred interactions between cell types in the RbV group. The color range is scaled to the maximum number of interactions between a pair of cell types in the RbV group.
